# Desmoid Tumor of the Anterior Abdominal Wall in Female Patients: Comparison with Endometriosis

**DOI:** 10.1155/2012/725498

**Published:** 2012-06-12

**Authors:** H. Krentel, G. Tchartchian, R. L. De Wilde

**Affiliations:** ^1^Department of Obstetrics and Gynecology, Hospital Eisenhüttenstadt, Friedrich-Engels-Str. 39, 15890 Eisenhüttenstadt, Germany; ^2^CEGPA, Av. Guardia Civil 135, San Borja, Lima, Peru; ^3^MIC Clinic Berlin, Kurstr. 11, 14129 Berlin, Germany; ^4^Department of Obstetrics and Gynecology, Pius Hospital, Georgstr. 12, 26121 Oldenburg, Germany

## Abstract

In female patients presenting a tumor of the lower abdominal wall especially after cesarian section, an endometriotic tumor as well as an aggressive desmoid tumor should be considered. Symptoms in correlation with the monthly period can facilitate the presurgical differentiation between endometriosis and fibromatosis. Ultrasound reveals the typical location of both tumors and its remarkable sonographic appearance. In the clinical practice, the desmoid fibromatosis of the lower abdominal wall is a very rare disease. We present a case of a 25-year-old pregnant and discuss diagnostic and therapeutic options by a PubMed literature review. With the knowledge of the prognosis of the desmoid fibromatosis and the respective treatment options including wait and see, complete surgical resection with macroscopically free margins and adjuvant approaches is essential to avoid further interventions and progression of the locally destructive tumor.

## 1. Introduction

Pregnant women with a cesarean section in their clinical history occasionally present a tumor of the lower anterior abdominal wall. In many cases, the tumor located close to the C-section scar appears in the second or third trimester of an actual pregnancy is palpable and temporarily causes pain. An irregular, inhomogeneous focus can be found in sonographic examination with the convex scan. Mostly the clinical diagnosis is endometriosis. The tumor resection as part of the C-section is the usual treatment. In this paper we present a desmoid fibromatosis of the lower abdominal wall in a young pregnant woman and discuss the treatment options of desmoid tumors in pregnancy and the differentiation in diagnosis and treatment between endometriosis and desmoid-type tumors considering the results of a literature review. The correct pre- and intraoperative diagnosis of this rare disease is the most important factor for the respective treatment and prognosis of the destructive desmoid fibromatosis. 

## 2. Case Presentation

In this paper we describe the case of a 25-year-old woman with 38 weeks of gestation and a symptomatic tumor of the lower abdominal wall. We supposed the tumor to be a scar endometriosis as the patient had a previous caesarian section and the tumor was located 2 cm cranial to the scar. Therefore, a preoperative diagnostic cytology has not been performed. The presurgical examination showed a dense and painful mass with a diameter of 3-4 cm. Ultrasound revealed a subfascial, intramuscular localization. The tumor did not disturb the uterus and the pregnancy. As a caesarian section has been planned, we decided to resect the tumor within the surgery. After successful child delivery and closure of the uterine tissue and the peritoneum, we found a dense, incompressible, grey tumor in the right rectus sheath with a local infiltration of the rectus muscle. A local complete resection with a macroscopically tumor-free margin was performed. The tumor size of not more than 3 cm allowed the resection without any functional limitations. A mesh reconstruction was not necessary. Surprisingly, an aggressive musculoaponeurotic fibromatosis was found in the histologic examination. The patient recovered without any complications or functional defects. We recommended clinical and sonographic controls of the tumor location. Since 2 years after surgical intervention, the patient is recurrence-free.

## 3. Discussion

The desmoid fibromatosis is characterized by local aggressive growth without any tendency of metastasis. These very rare tumors can develop in any musculo-aponeurotic structure, and they can be found in all regions of the human body. The commonest site of presentation in case of abdominal wall desmoids seems to be the infraumbilical rectus sheath. The clinical behaviour and the prognosis of the desmoids is very diverse and depends on the anatomic location and the proximity to vitally important organs. A correlation with the familiar intestinal polyposis could be shown [1]. Approximately 10–25% of the patients with polyposis present intra- or extra abdominal desmoid tumors [2]. Supposed risk factors of desmoids are previous surgical interventions, pregnancy and hormonal treatment with estrogen. In the presented case, the local tissue trauma of the cesarean section in the clinical history of the patient is one possible risk factor of the disease. The estrogen dependence of the tumors explains the stimulation of their growth by estrogen high flux during pregnancy. Michopoulou et al. published the case of a 37-year-old pregnant presenting a fast growing desmoid of the abdominal wall, finally reaching a diameter of 20 cm [3]. Viriyaroj et al. [4] found an aggressive fibromatosis of almost 5 kg in the lower abdominal wall of a 17-year-old pregnant. 8 months after resection, the patient did not show any recurrence of the disease. Cormio et al. reported a recurrence-free period of 30 month after surgical resection of a pelvic fibromatosis without any adjuvant treatment [5]. The recurrence of desmoid tumors ranges from 20 to 60% in large retrospective studies. However, in many cases, a recurrence of the aggressive tumors after resection is described [6]. In a retrospective review of 151 patients who underwent a macroscopically complete resection of desmoid tumors, Huang et al. showed a local recurrence rate of 20.5%. Admission status, gender, tumor size, margin status, location, and number of tumors are predictive factors of local recurrence [7]. Salas et al. showed three unfavorable prognostic factors: age less than 37 years, tumor size >7 cm, and extra-abdominal tumor location [8]. A clear difference in progression free survival was demonstrated according to the number of prognostic factors present. Kumar et al. described a recurrence rate of 25% in a group of 32 patients. The most important factor for recurrence was a tumor size of >5 cm [9]. Bertani et al. described a recurrence-free followup period of 55 months in 14 patients with disease-free margins of >1 cm in intraoperative frozen section [10]. Depending on the tumor size, a reconstruction of the abdominal wall defect with meshes after resection can be necessary [11]. The early detection of a desmoid and the respective treatment of a smaller tumor can prevent large defects and the mesh reconstruction. However, other authors report that there is no correlation between incomplete resection and recurrency rate. Arrest of growth or even regression is a frequent occurrence even in the absence of any specific treatment. And the local tissue trauma by surgery might further accelerate the evolution of the disease. Salvi et al. therefore recommend a conservative clinical control [12]. Barbier et al. prefer a wait and see strategy in large tumors, when the resection would cause an important functional and esthetic limitation [13]. Fiore et al. reported a 5-year progression-free survival in almost 50% of a group of 83 patients receiving a wait and see strategy [14]. Anyway, other publications show adjuvant therapeutic approaches to avoid tumor growth and recurrence after surgical resection. Seoud et al. reported about the case of a 32-year-old female with a large pelvic desmoid tumor, who underwent adjuvant radiation after complete resection. A recurrence-free period of 6 years could be shown with this treatment [15]. However, it remains unclear if a wait and see strategy or a surgical treatment without adjuvant radiation would have caused another result. Huang et al. and Kumar et al. [7, 9] for example could not show any significant benefit over local recurrence rate with adjuvant radiation. Sportiello et al. reported a case of a desmoid developing in pregnancy with recurrence despite of twice complete resection and adjuvant radiation. Although desmoids normally do not express estrogen receptors [12], the treatment with tamoxifen finally caused the complete regression of the tumor [16]. Chao et al. also described the complete regression of a tumor recurrence by tamoxifen [17]. Maseelall et al. reported the successful postoperative therapy of a pelvic fibromatosis with Toremifene. The antiestrogenic treatment allowed a recurrence-free period of nine years until the menopause of the pacient [18]. Constantinidou et al. published the successful use of chemotherapy in advanced aggressive fibromatosis with no response to hormonal treatment [19]. In another actual publication the possibility of a therapy with tyrosine kinase inhibition in inoperable tumors is described [20]. Skubitz et al. showed the effectiveness of sunitinib in imatinib-resistant desmoid fibromatosis of the lower extremity [21]. A clear therapeutic approach in all cases of desmoid tumors cannot yet be preferred, and further studies are needed to describe the outcome of the different treatment options. In pregnancy, the desmoid tumor of the lower abdominal wall, especially in the area of a cesarean section scar, can be confound with a scar endometriosis. A clear clinical diagnosis of the tumors origin and a differentiation to endometriosis by palpation and ultrasound are not possible. The intramuscular location of the tumor in ultrasound is an indicator for a desmoid tumor. While desmoids in almost all cases are located close to a muscular aponeurosis, the endometriotic tumors of the abdominal wall can be found most frequently in the subcutaneous region. In the actual literature, only 18 case reports about endometriosis exclusively located in the rectus muscle sheath can be found [22]. A typical sonographic feature of all intramuscular desmoids is the spindle-shaped margin at the tumor ends when scanned along the long axis of the affected muscle. The desmoids arising from the fascia show an irregular shape [23]. However, endometriosis as well as desmoids of the abdominal wall can develop without previous surgery. Granese et al. and Gourgiotis et al. described two cases of endometriotic tumors in the rectus muscle in patients who did not undergo any surgical interventions [24, 25]. A tomography or MRI can be especially helpful in the differentiation of desmoids from endometriosis [26]. Sauter et al. recently described the monitoring of the response of desmoids to a therapy with imatinib using MRI [27]. A presurgical fine-needle aspiration cytology can help to avoid mistakes in the approach of the abdominal wall tumors [28]. The correlation of tumor pain and the patients menstruation and the correlation of tumor volume and menstruation can be indicators for endometriotic tumors. Especially in pregnancy, the differentiation between endometriosis and desmoid tumor can help to find the indicated treatment options. The wait and see policy is a rational treatment option in both cases as the postpartum drop at estrogen level could lead to significant tumor regression in desmoids and endometriosis. In large tumors, the surgical intervention can be realized without functional limitations after tumor regression. In our case, the tumor resection followed the successful delivery during a preplanned cesarean section. Embryo safety has to be considered thinking about therapeutic approaches during pregnancy.

## 4. Conclusion

The wait and see strategy is a rational treatment option in case of desmoid tumors in pregnancy. Depending on the risk factors age, tumor size, and location, the different treatment approaches should be considered after successful delivery. The postpartum drop of estrogen level could lead to a tumor regression, which could avoid a mutilating surgery. Regular controls with ultrasound or MRI should show a progression or recurrence of a desmoid tumor before the appearance of symptoms. The surgical intervention has to be the first choice in desmoids close to vitally important organs or very large tumors compressing the uterus. The adjuvant approach with radiation, hormonal therapy, and chemotherapy should be considered individually. A targeted therapy with tyrosin kinase inhibition can be a further treatment option. In case of a tumor of the lower abdominal wall in female patients, the desmoid can be confound with endometriosis. Ultrasound and a fine-needle aspiration cytology can help to find the presurgical diagnosis and to avoid surprising results.
